# Research Training of Future Leaders in Radiology: A Pilot International Outreach Educational Program

**DOI:** 10.7759/cureus.109288

**Published:** 2026-05-20

**Authors:** Andrea S Doria, Jennifer R McKinney, Aleena Malik, Paulo Helito, Marcelo Bordalo Rodrigues, Ivan Diamond, George Tomlinson, Christopher Meaney, Carina De Micheli, Jonathan J Chan, Ricardo Faingold, Giovanni Cerri, Claudia Da Costa Leite

**Affiliations:** 1 Diagnostic and Interventional Radiology, The Hospital for Sick Children, Toronto, CAN; 2 Medical Imaging, University of Toronto, Toronto, CAN; 3 Radiology, Instituto de Radiologia, Hospital das Clínicas da Faculdade de Medicina da Universidade de São Paulo (HCFMUSP), São Paulo, BRA; 4 Radiology, Instituto de Ortopedia e Traumatologia, Hospital das Clínicas da Faculdade de Medicina da Universidade de São Paulo (HCFMUSP), São Paulo, BRA; 5 Clinical Epidemiology and Health Care Research, University of Toronto, Toronto, CAN; 6 Family and Community Medicine, University of Toronto, Toronto, CAN

**Keywords:** brazil, canada, education, radiology, research

## Abstract

Objective

This study aimed to determine the short-term effects of a research methodology course (intervention) on knowledge enhancement of participants from economically emerging (cohort 1) and economically developed (cohort 2) countries; and to determine the long-term publication trajectory of course participants from the time of intervention (timepoint 1 (T1)) to 10 years post-course (timepoint 2 (T2)).

Participants/setting

Residents/fellows/junior staff from academic institutions of cohorts 1 and 2 completed a four-day research course divided into research design (M1) and statistics (M2) modules.

Intervention

A 30-item multiple-choice pre-test was provided at the beginning and end of the course for M1 and M2. Pre-/post-test scores assessed knowledge enhancement. A PubMed search (1966-2024) was conducted to evaluate the number, impact factor (IF), and authorship of publications until T1 and T2.

Results

In cohort 1, 21 (100%) participants (male-to-female ratio: 11:10; six residents and 15 fellows/junior staff) aspired to an academic career; in cohort 2 (male-to-female ratio: 12:7), 12/19 (63%) (seven residents and five fellows/junior staff) did. At T1, differences were noted between pre- and post-test scores for cohort 1 in M1 (p=0.0005) and M2 (p=0.001), but only for M2 (p=0.01) for cohort 2. For aspiring academicians, whereas the mean number of publications/person was similar between cohort 1 (N=1.1) and cohort 2 (N=2.75) at T1 (p=0.12), it was higher for cohort 2 (N=27.4 vs. N=8.95) at T2 (p=0.03), with similar mean IF of publications for cohorts 1 and 2 at T1 and T2 (p>0.05).

Conclusion

In the short term, radiology trainees from both countries benefited from the educational research program, particularly in statistics. Over a decade, whereas the quality (IF) of publications remained stable for academicians of both countries, the publication output/person remained stable for cohort 2 participants but declined for cohort 1 participants.

## Introduction

Radiology is a rapidly growing clinical specialty that has a key role in decision-making toward patient care [1]. Despite the outstanding technological development of the radiological sciences in recent years, the scientific productivity and research leadership of this specialty have been below the levels achieved by other clinical specialties [2]. There are several reasons for this imbalance. Chief among them is the lack of well-trained radiologists in the research field [3] who could pursue academic careers playing a "bridging" role in health research and clinical practice [4-6]. 

To fill this gap in the production of high-quality research on topics of interest, education in research of the new generation of leaders in radiology plays a key role. There is evidence of the value of lecture- and interactive-based introductory radiology curriculum on trainees' preparedness for implementing their performance and skills on specific aspects of their training [7,8]. This is needed not only in selected countries but globally [9-11]. Very few academic programs in developing countries have specific training in research during residency and fellowship [12]. 

Collaborative research between radiology departments of different nations enables the expansion of the potential for investigation of the entire spectrum of severity of diseases from minimal to advanced pathological changes. With the economic "boom" that took place in several developing countries in the last decade, imaging centers of excellence have started to flourish in these countries. Some of these centers possess the necessary resources and infrastructure to conduct high-quality research but have a track record of lower academic productivity compared to academic centers of developed countries due to regional and administrative issues.

An example of such an imaging center of excellence in Latin America is Universidade de São Paulo (USP) in Brazil. USP is one of the largest academic institutions in Latin America with a leading role in the development of guidelines in education and research in Brazil and other countries in Latin America. The Department of Radiology at USP holds one of the largest residency/fellowship programs in Brazil (approximately 24 residents and 22 fellows per year). 

The first step in the process of integration of research in radiology between academic institutions of developed and developing countries is the implementation of research training of residents and fellows who will be the future workforce of the specialty. The second step is the development of an active and sustainable platform of collaborative research between institutions using this project as the stepping stone for further education in the research of trainees in radiology.

The Department of Medical Imaging at the University of Toronto (UofT) in Canada has the largest residency (11 new residents per year) and fellowship (over 60 new fellows per year) programs in Canada. UofT has been conducting an annual research course for residents and fellows of radiology since 2006. The course was initially offered over an entire academic year; however, since 2008, it has been offered in the format of a four-day workshop that is part of the radiology residency curriculum at UofT. Both UofT (Canada) and USP (Brazil) have common interests toward building capacity in research education for radiology trainees, and investigators of the two programs have a track record of prior research collaborations. 

This study had twofold purposes: (1) to determine the short-term effects of an intervention, a research methodology course, on knowledge enhancement of participants (mean difference in pre-/post-test scores) of an economically emerging country, Brazil, and of a developed country, Canada, and (2) to evaluate the publication trajectory during the follow-up of course participants at the time of the intervention (timepoint 1) and the trajectory recorded 10 years post-course (timepoint 2). The primary outcome of this study was short-term knowledge enhancement, defined as the difference between pre- and post-test scores following the intervention. Secondary outcomes included long-term academic productivity metrics, specifically the number of publications, impact factor (IF) of publications, and authorship patterns assessed at timepoints 1 and 2.

## Materials and methods

Following approval from the Research Ethics Board (REB) of the Hospital for Sick Children (approval number: 4189) and the Research Ethics Committee of the Hospital das Clínicas da Faculdade de Medicina da Universidade de São Paulo (HCFMUSP) (approval number: 3.043.745), a retrospective study was conducted at the University of Toronto (UofT) (Toronto, Canada) and in the Instituto de Radiologia (INRAD) (Universidade de São Paulo (USP), São Paulo, Brazil).

Procedure

A four-day research course was administered to radiology residents and fellows/junior staff under the guidance of Canadian investigators at UofT. The course provided basic concepts and practical exercises on research methodology and statistics applied to the radiological sciences to participants. Its goal was to help attendees improve the research design of their projects during residency, during fellowship, or as part of academic work as well as to motivate them to go deeper into research ideally pursuing further graduate education in the research field, if feasible. The course was administered on two separate occasions, first at a UofT (Toronto, Canada) educational facility in October 2014 and then at a USP (São Paulo, Brazil) educational facility in December 2014. The content of the curriculum of the course that was delivered face-to-face to attendees of the two institutions was similar and is shown in Appendix A. Four of the 11 (36%) lecturers and tutors who participated in Canada's course also participated in Brazil's course. All of the lecturers had graduate degrees (MSc or PhD) in epidemiology or statistics. The selection criteria of participants at USP were to be residents of any year and fellows (equivalent to PGY5 residents in Canada) and junior staff in their first two years with an interest in research. In contrast to Canada, where the radiology residency program has a four-year length (after a one-year internship), in Brazil, it has a three-year length (with no internship period after medical school graduation). The optional fourth year of radiology training in Brazil is called "fellowship". Junior staff of Brazil were in their first or second year post-three years of residency being equivalent in number of years of training to fellows in Canada. Therefore, for the purpose of this study, USP junior staff within the first two years of post-residency completion were eligible for participation. The selection criteria of participants at UofT were to be PGY3 residents (the course was offered as part of their residency curriculum) and fellows (of any radiology subspecialty) and junior staff with an interest in research. No formal research course or structured training had been available for USP participants during their training prior to this study. For the short-term knowledge enhancement analysis, scores of participants of a given module were excluded if they failed to take both pre- and post-tests for that module. 

The course was divided into two parts: module 1, research design methodology, was delivered on the first two days, followed by module 2, statistics, delivered on the subsequent two days. Objectives of individual educational sessions are described in Appendix B. Trainees were required to complete 30 multiple-choice questions, each with four choices (a-d), as a pre-test at the beginning of each module, followed by a post-test with identical questions at the end. The 30-item multiple-choice assessment tool was developed by investigators with expertise in epidemiology and biostatistics and was designed to reflect core competencies in research methodology and statistical analysis relevant to radiology trainees. While the instrument was not formally validated externally, it was internally reviewed and standardized across both cohorts to ensure consistency in content and level of difficulty. The difference between pre-test and post-test scores served as an indicator of knowledge enhancement. One participant from Brazil attended the same course in Canada (in October 2014) and in Brazil (in December 2014). His pre- and post-test scores were not included in the calculations of mean (standard deviation (SD)) and % score improvement of Brazil participants. Instead, they were reported separately (Table 1).

**Table 1:** Mean (standard deviation (SD)) pre- and post-test scores (difference and interval score change (%)) for USP, Brazil, and UofT, Canada Numbers shown in italics indicate significant differences between pre- and post-test scores. USP: Universidade de São Paulo; UofT: University of Toronto

Module	Country	Participants	Pre-test mean (SD) scores	Post-test mean (SD) scores	Difference (SD)	P-value	Interval score change (%, SD, range)
Module 1	USP (Brazil)	Total (n=18; 2 excluded, 1 dual)	52.0 (11.4)	64.7 (8.5)	12.9 (12.1)	0.0005	30% (40%, -10% to 90%)
		Residents (n=6)	53.3 (14.1)	67.2 (7.1)	13.9 (14.7)	0.07	30% (40%, -10% to 90%)
		Fellows/junior staff (n=12)	51.4 (10.4)	63.9 (9.4)	12.5 (14.2)	0.005	30% (50%, -20% to 160%)
	-	Junior staff Brazil (dual timepoint test) (n=1)	76.7	80.0	3.3	-	4%
	UofT (Canada)	Total (n=19)	61.1 (15.8)	67.0 (11.5)	6.0 (9.2)	0.19	10% (23%, -20% to 80%)
		Residents (n=13)	65.9 (8.9)	69.7 (8.5)	3.8 (8.4)	0.27	10% (14%, -20% to 30%)
		Fellows (n=6)	50.6 (22.6)	61.1 (15.6)	10.6 (9.9)	0.37	30% (30%, -4% to 80%)
	-	Junior staff Brazil (dual timepoint test) (n=1)	66.7	66.7	0.0	-	0%
Module 2	USP (Brazil)	Total (n=17; 4 excluded, including dual test)	39.6 (10.3)	53.8 (10.7)	10.7 (12.2)	0.001	40% (40%, -20% to 140%)
		Residents (n=6)	40.6 (7.4)	49.4 (10.6)	8.9 (10.7)	0.13	20% (30%, -10% to 60%)
		Fellows/junior staff (n=11)	39.1 (11.8)	52.1 (8.1)	13.0 (12.9)	0.007	50% (50%, -20% to 140%)
	-	Junior staff Brazil (dual timepoint test) (n=1)	76.7	73.3	-3.3	-	-4%
	UofT (Canada)	Total (n=19)	59.8 (11.0)	68.8 (9.4)	8.9 (9.1)	0.01	20% (18%, -20% to 50%)
		Residents (n=13)	61.8 (9.4)	71.8 (8.3)	10.0 (7.5)	0.008	20% (14%, 0% to 40%)
		Fellows (n=6)	55.6 (13.9)	62.2 (8.9)	6.7 (12.5)	0.35	20% (27%, -20% to 40%)
	-	Junior staff Brazil (dual timepoint test) (n=1)	73.3	76.7	3.3	-	4%

Evaluation forms, which included a multiple-choice survey and open-ended questions, were administered to trainees of both centers at the end of the course upon completion of the post-tests (Appendix C). The multiple-choice survey consisted of several statements which trainees ranked on a scale from 1, meaning poor/strongly disagree, to 5, meaning excellent/strongly agree. Topics included interest in radiology research, protected time for research, and pursuing an academic career as a clinician-scientist. Attendance was taken at the beginning of each day, and only radiology residents and fellows who attended all four days and completed both the pre- and post-tests were included in the study for consistency purposes.

Retrospectively, a PubMed search starting in January 1966 was conducted. The search strategy included combinations of last names and first and middle initials for each participant. To improve accuracy and reduce ambiguity in author identification, results were cross-verified using institutional affiliations, geographic identifiers, and known co-authorship patterns. In cases of uncertainty, manual review of publication records was performed to ensure correct attribution. We used an interval of time (up to timepoint 1, December 2014, and up to timepoint 2, June 2024, for all participants of both countries) to determine the number and impact (IF) of publications of each participant at the time of the intervention. Scientific presentations, abstracts, and book chapters were not included. Only journal articles written in English and indexed in PubMed were included. Order of authorship was also examined. Afterwards, the results of this study were discussed with the USP leadership responsible for the academic education of their residents and fellows to best understand the rationale for them. 

Statistical analysis

Score difference between tests administered before or after the course was defined as follows: $\text{Post-test score}-\text{Pre-test score}$. Interval score change (%) was defined as follows: $\left[\left(\text{Post-test score}-\text{Pre-test score}\right)/\text{Pre-test score}\right]\times100$.

We hypothesized that a minimum of 20% increase or a 5-point score difference would be achieved in ≥80% of radiology trainees who actively participated in the research course. Knowledge enhancement scores were also expected to be similar (±5% of SD) in the same year groups of USP and UofT trainees. These hypotheses were defined a priori to evaluate the primary outcome of short-term knowledge enhancement, while analyses of publication number, IF, and authorship patterns were conducted as secondary, exploratory outcomes.

Differences between pre- and post-tests for each category of participants were evaluated by paired Student's t-tests. Differences between mean pre-test scores from Brazilian and Canadian participants and between mean post-test scores from these participants were assessed by unpaired Student's t-tests. 

The mean number of publications/person, IF/person, and number of first-author publications/person were compared between Brazilian and Canadian participants and between different categories of Canadian participants using unpaired Student's t-tests. Analyses of IF included only those with publications.

A separate descriptive analysis was performed for residents and fellows/junior staff groups. Statistical analysis was performed with a StatPlus (AnalystSoft, Alexandria, Virginia, United States)/Excel 2013 package (Microsoft Corporation, Redmond, Washington, United States). Two-tailed p-values of less than 0.05 were taken to be statistically significant.

This structured distinction between primary and secondary outcomes ensured alignment between study objectives, statistical analysis, and interpretation of results.

## Results

Course attendees included six (28.6%) residents and 15 (71.4%) fellows/junior staff from USP and 13 (72.2%) residents and five (27.8%) fellows from UofT. No junior staff from UofT attended the course. There were 11 (52.4%) males and 10 (47.6%) females in the Brazilian cohort and 12 (66.7%) males and six (33.3%) females in the Canadian cohort.

All 21 USP candidates were academician candidates, of which 10 (47.6%) USP trainees desired to pursue a clinician-scientist career (with 50% of protected time) if this opportunity would be available, while the remaining 11 (52.4%) were interested in an academic career (with 20% protected time). Among 18 UofT trainees, four (22.2%) desired to be clinician-scientists (with 50% protected time), eight (44.4%) were interested in an academic career (with 20% protected time), and the remainder were not interested in pursuing an academic career. Therefore, 66.7% of UofT participants were academician candidates. None of the participants of either cohort was pursuing or already had an MSc or PhD degree. 

Concerning the knowledge enhancement analysis of the study, two USP participants had their scores excluded in module 1 (total assessed, 19 out of 21, 90.5%) and three in module 2 (total assessed, 18 out of 21, 85.7%). No UofT participants had scores excluded (Table 1).

Short-term knowledge enhancement

Whereas USP trainees demonstrated significant knowledge enhancement for both modules 1 (p=0.0005) and 2 (p=0.001) (Table 1), UofT trainees showed significant knowledge enhancement for module 2 (p=0.01), but no substantial difference between pre- and post-test scores for module 1 (p=0.19). Whereas the mean interval score changes for modules 1 and 2 for USP trainees were 30% and 40%, respectively, they were only 10% and 20% for UofT trainees, respectively (Table 1). Hence, although USP's average interval score changes for both modules 1 and 2 and UofT's average score changes for module 2 met the hypothesized 20% score change improvement, UofT's average score changes for module 1 did not.

UofT trainees had higher pre-test scores, respectively (module 1, p=0.05; module 2, p=0.000002), than USP trainees. Although participants of both countries have reached a similar level of competence for module 1 by the end of the course, as shown by similar post-test scores obtained (p=0.55), post-test score differences were noted between participants of both countries for module 2 (p=0.00002), for both residents and fellows/junior staff (Table 2). 

**Table 2:** Differences in background knowledge of participants (based on pre-test scores) and final post-test scores achieved after the intervention (research course) in participants of both centers Numbers shown in italics indicate significant differences between pre- and post-test scores. Excluded junior staff with dual participation in the Canada and Brazil cohorts. USP: Universidade de São Paulo; UofT: University of Toronto

Module	Group	USP (Brazil) pre-test mean (SD)		UofT (Canada) pre-test mean (SD)		Pre-test p-value	USP (Brazil) post-test mean (SD)	UofT (Canada) post-test mean (SD)	Post-test p-value
Module 1	Total	n=18 (2 excluded, 1 dual)	52.0 (11.4)	n=19	61.1 (15.8)	0.054	64.7 (8.5)	67.0 (11.5)	0.55
Module 1	Residents	n=6	53.3 (14.1)	n=13	65.9 (8.9)	0.09	67.2 (7.1)	69.7 (8.5)	0.51
Module 1	Fellows/junior staff	n=12	51.4 (10.4)	n=6	50.6 (22.6)	0.93	63.9 (9.4)	61.1 (15.6)	0.70
Module 2	Total	n=17 (4 excluded, including dual test)	39.6 (10.3)	n=19	59.8 (11.0)	0.000002	53.8 (10.7)	68.8 (9.4)	0.00002
Module 2	Residents	n=6	40.6 (7.4)	n=13	61.8 (9.4)	0.0001	49.4 (10.6)	71.8 (8.3)	0.001
Module 2	Fellows/junior staff	n=11	39.1 (11.8)	n=6	55.6 (13.9)	0.04	52.1 (8.1)	62.2 (8.9)	0.04

It should be noted that both residents and fellows of UofT performed better in pre- and post-tests of module 2 than trainees of USP in corresponding categories (pre-test scores: p=0.0001 for residents and p=0.04 for fellows/junior staff; post-test scores: p=0.001 for residents and p=0.04 for fellows/junior staff). No such difference between residents and fellows/junior staff of the two institutions was noted for pre-or post-test scores of module 1 (Table 2).

Mid-term publication trajectory of participants

At timepoint 1, the USP and UofT aspiring academicians had similar mean number of publications/person (mean/SD for USP: 1.09/1.37; mean for UofT: 2.75/2.55 (p=0.12)). Nevertheless, over the timeframe of 10 years, the UofT group progressed towards generating a significantly higher lifetime publication output/person than the USP group (mean/SD for USP: 8.95/10.43; mean for UofT: 27.38/19.58 (p=0.03)). This observation was not noted for the groups of USP and UofT aspiring clinician-scientists which demonstrated a similar publication output/person rate for both groups at both timepoints (Table 3).

**Table 3:** Mean (standard deviation (SD); range) number of lifetime publications/person per timepoint, mean impact factor of lifetime publications per timepoint, and mean lifetime number of papers as primary and second author/person for different categories of expected careers of participants of Brazil and Canada Numbers shown in italics indicate significant differences between pre- and post-test scores. USP: Universidade de São Paulo; UofT: University of Toronto

Country	USP (Brazil) aspiring clinician-scientists (n=10/21)	USP (Brazil) aspiring academicians (n=21/21)	UofT (Canada) aspiring clinician-scientists (n=4/18)	UofT (Canada) aspiring academicians (n=8/18)	UofT (Canada) non-aspiring academicians (n=10/18)	P-value
Mean number (SD, range) of lifetime publications/person at timepoint 1 (time of course)	1.70 (1.70, 0-5)	-	4.25 (2.63, 2-8)	-	-	0.15
Mean number (SD, range) of publications at timepoint 2 (10-year follow-up)	15.6 (11.82, 3-43)	-	25.25 (20.17, 11-55)	-	-	0.42
Mean number (SD, range) of publications/person at timepoint 1 (time of course)	-	1.09 (1.37, 0-5)	-	2.75 (2.55, 0-8)	-	0.12
Mean number (SD, range) of publications at timepoint 2 (10-year follow-up)	-	8.95 (10.43, 0-43)	-	27.38 (19.58, 8-58)	-	0.03
Mean number (SD, range) of publications/person at timepoint 1 (time of course)	-	-	-	2.75 (2.55, 0-8)	1.30 (1.64, 0-4)	0.19
Mean number (SD, range) of publications at timepoint 2 (10-year follow-up)	-	-	-	27.38 (19.58, 8-58)	3.6 (3.13, 0-10)	0.01
Mean number (SD, range) of publications/person at timepoint 1 (time of course)	-	1.09 (1.37, 0-5)	-	-	1.30 (1.64, 0-4)	0.74
Mean number (SD, range) of publications at timepoint 2 (10-year follow-up)	-	8.95 (10.43, 0-43)	-	-	3.6 (3.13, 0-10)	0.04
Mean (SD, range) publication output impact factor/person at timepoint 1 (time of course)	1.51 (1.11, 0.395-3.631)	-	4.40 (2.95, 1.180-8.136)	-	-	0.12
Mean (SD, range) impact factor/person at timepoint 2 (10-year follow-up)	2.78 (0.80, 1.81-4.55)	-	4.43 (1.87, 2.54-6.98)	-	-	0.19
Mean (SD, range) impact factor/person at timepoint 1 (time of course)	-	1.84 (1.37, 0.395-4.991)	-	3.88 (2.54, 1.180-8.136)	-	0.11
Mean (SD, range) impact factor/person at timepoint 2 (10-year follow-up)	-	2.85 (1.13, 1.27-5.04)	-	4.21 (1.65, 1.63-6.98)	-	0.06
Mean (SD, range) impact factor/person at timepoint 1 (time of course)	-	-	-	3.88 (2.54, 1.180-8.136)	2.93 (2.52, 0.735-7.216)	0.55
Mean (SD, range) impact factor/person at timepoint 2 (10-year follow-up)	-	-	-	4.21 (1.65, 1.63-6.98)	3.68 (2.03, 0.74-7.22)	0.56
Mean (SD, range) impact factor/person at timepoint 1 (time of course)	-	1.84 (1.37, 0.395-4.991)	-	-	2.93 (2.52, 0.735-7.216)	0.40
Mean (SD, range) impact factor/person at timepoint 2 (10-year follow-up)	-	2.85 (1.13, 1.27-5.04)	-	-	3.68 (2.03, 0.74-7.22)	0.28
Mean lifetime number of papers (SD, range) as first author/person	3.80 (3,88, 0-11)	-	5.75 (2.38, 4-9)	-	-	0.28
Mean number of papers (SD, range) as second author/person	3.30 (4.79, 0-16)	-	5.25 (5.5, 0-10)	-	-	0.56
Mean number of papers (SD, range) as first author/person	-	2.611 (3.22, 0-11)	-	5.13 (2.95, 1-9)	-	0.07
Mean number of papers (SD, range) as second author/person	-	2.35 (3.84, 0-16)	-	6 (4.38, 0-12)	-	0.07
Mean number of papers (SD, range) as first author/person	-	-	-	5.13 (2.95, 1-9)	1.57 (1.27, 0-4)	0.01
Mean number of papers (SD, range) as second author/person	-	-	-	6 (4.38, 0-12)	1.57 (1.27, 0-3)	0.03
Mean number of papers (SD, range) as first author/person	-	2.611 (3.22, 0-11)	-	-	1.57 (1.27, 0-4)	0.26
Mean number of papers (SD, range) as second author/person	-	2.35 (3.84, 0-16)	-	-	1.57 (1.27, 0-3)	0.46

Aspiring academicians from UofT showed a statistically significant greater lifetime publication output rate in relation to USP participants at timepoint 2 (p=0.03), and aspiring UofT academicians also produced a greater lifetime publication output rate in relation to UofT non-academicians at timepoint 2 (p=0.01). Aspiring USP academicians produced a greater lifetime publication output rate in relation to UofT non-academicians (p=0.04) at timepoint 2. There was no statistically significant difference in the lifetime publication output rate between other categories of participants (aspiring clinician-scientists of both centers and aspiring academicians of USP). Of note, the lifetime publication output rates of aspiring and non-aspiring UofT academicians were not statistically different at timepoint 1 (p=0.19), but it was statistically significant at T2 (p=0.01). This was also the case for the lifetime publication output of USP aspiring academicians and non-aspiring UofT academicians which was not significantly different at timepoint 1 (p=0.74), but the USP aspiring academicians had a significantly higher mean number of lifetime publications at timepoint 2 (p=0.04).

The mean (SD) IF of lifetime publications of USP and UofT aspiring clinician-scientists was not different at either timepoint when compared to one another (Table 3). Likewise, they were not different between aspiring and non-aspiring Canadian academicians at either timepoint. Nevertheless, both Brazilian aspiring clinician-scientists (from a mean of 1.51 (SD, 1.11) to a mean of 2.78 (SD, 0.80); p=0.03) and aspiring academicians (from a mean of 1.84 (SD, 1.37) to a mean of 2.85 (SD, 1.13); p=0.047) had a 10-year interval increase of IF of their publications, while no such statistically significant increase was noted in any of the other mean IFs of participants of the two countries (Table 3). Concerning interval changes, a greater mean number of lifetime publications in timepoint 2 was observed for USP aspiring academicians and clinician-scientists and the UofT aspiring academicians in comparison with timepoint 1. Meanwhile, the mean IF of publications in timepoint 2 was significantly increased for both the Brazilian aspiring clinician-scientists and academicians (p=0.027 and p=0.47, respectively), but not for any of the UofT groups. Tables 4-6 provide raw data for the mean IF of lifetime publications at timepoints 1 and 2.

**Table 4:** Mean IF of lifetime publications between timepoints 1 (baseline) and 2 (10 years later) for participants of the University of Toronto, Canada "-" indicates no publications at timepoint. IF: impact factor; T1: timepoint 1; T2: timepoint 2

University of Toronto	Average IF T1	Average IF T2
Aspirants to academicians (N=8)		
Fellow 1	-	3.77
Fellow 2	1.18	1.63
Resident 4	3.22	2.54
Resident 6	8.14	4.42
Resident 9	5.09	6.98
Resident 11	-	4.33
Fellow 3	1.68	4.56
Fellow 4	3.96	5.42
Aspirants to clinician-scientists (N=4)		
Fellow 2	1.18	3.77
Resident 4	3.22	2.54
Resident 6	8.14	4.42
Resident 9	5.09	6.98
Non-academicians (N=10)		
Fellow 5	-	6.45
Resident 1	1.57	2.34
Resident 2	0.74	0.74
Resident 3	-	3.63
Resident 5	2.4	2.63
Resident 7	-	-
Resident 8	2.76	2.76
Resident 10	-	4.03
Resident 12	-	3.33
Resident 13	7.22	7.22

**Table 5:** Mean IF of lifetime publications between timepoints 1 (baseline) and 2 (10 years later) for participants of Universidade de São Paulo, Brazil "-" indicates no publications at timepoint. IF: impact factor; T1: timepoint 1; T2: timepoint 2

Universidade de São Paulo	Average IF T1	Average IF T2
Aspirants to clinician-scientists (N=10)		
Resident 1	3.63	4.55
Fellow 3	2.07	2.31
Resident 3	-	2.85
Fellow 5	0.84	1.81
Resident 5	0.88	2.64
Junior staff 4	-	2.88
Junior staff 5	0.9	1.97
Junior staff 6	0.4	3.09
Junior staff 8	1.84	2.28
Fellow 6	-	3.37

**Table 6:** Mean IF of lifetime publications between timepoints 1 (baseline) and 2 (10 years later) for all participants of both centers (University of Toronto, Canada, and Universidade de São Paulo, Brazil) "-" indicates no publications at timepoint. IF: impact factor; T1: timepoint 1; T2: timepoint 2

University of Toronto and Universidade de São Paulo	Average IF T1	Average IF T2
Aspirants to academicians (N=21)		
Fellow 1	1.74	1.67
Fellow 2	-	-
Fellow 3	2.07	2.31
Fellow 4	-	5.04
Fellow 5	0.84	1.81
Fellow 6	-	3.37
Resident 1	3.63	4.55
Resident 2	4.99	3.4
Resident 3	-	2.85
Resident 4	-	-
Resident 5	0.88	2.64
Resident 6	-	1.27
Junior staff 1	2.79	4.48
Junior staff 2	-	-
Junior staff 3	1.33	3.6
Junior staff 4	-	2.89
Junior staff 5	0.9	1.97
Junior staff 6	0.4	3.09
Junior staff 7	0.67	1.28
Junior staff 8	1.84	2.28
Junior staff 9	-	-

While UofT aspiring academicians had a higher number of first- and second-author publications/person than non-academicians at UofT (p=0.01 and p=0.03, respectively), no differences were noted in the mean number of first- and second-author papers between any of the other groups (Table 3). 

No gender differences were noted for the number of publications/person or IF of publications at any timepoint or for the number of first-author publications in either country.

## Discussion

Our study demonstrates that Brazilian radiology participants showed superior short-term knowledge enhancement, represented by interval score changes following the proposed intervention (research course modules 1 and 2), compared to Canadian trainees (Table 1). While aspiring academicians from Brazil significantly increased their mean number of publications and mean IF between timepoints 1 and 2 (Table 3), their lifetime publication output rate was lower than that of Canadian academicians at timepoint 2. Similarly, both the mean publication number and mean journal IF of publications of USP aspiring clinician-scientists were significantly greater in timepoint 2 in relation to timepoint 1. However, no significant difference was observed in the mean number of publications or the mean journal IF between the two timepoints for UofT aspiring clinician-scientists. These findings suggest that although Brazilian academicians increased their publication rate and IF over time, they had lower overall productivity than Canadian academicians at timepoint 2. This indicates that radiology trainees in Brazil initially acquired research knowledge effectively, but this did not translate into comparable long-term academic productivity. Importantly, the observed differences in long-term academic productivity between cohorts should be interpreted with caution. Given the observational design of the long-term follow-up and the absence of adjustment for potential confounding variables, these findings represent associations rather than causal relationships. Structural and contextual factors, including institutional research infrastructure, availability of protected research time, access to funding and incentives, and differing academic expectations, likely played a substantial role in shaping long-term publication outcomes and were not controlled for in this study. This study should be considered a pilot model demonstrating the feasibility and potential impact of structured international research training programs in radiology.

UofT aspiring academicians had a greater mean number of lifetime publications at timepoint 2 than UofT non-aspiring academicians (Table 3). They also demonstrated a higher mean number of first-author publications compared to UofT non-aspiring academicians. Interestingly, no significant differences in first- or second-author publication numbers were found when comparing Brazilian and Canadian groups. This underscores the need to incentivize research leadership among trainees and to develop curricula that encourage project ownership by junior staff.

The research course content of modules 1 and 2 assessed three domains: (1) baseline knowledge, represented by pre-test scores reflecting prior research training; (2) cumulative knowledge, represented by post-test scores encompassing both baseline and acquired knowledge; and (3) gradient score, calculated as $\text{Average interval score change (\%)}=\left[ \text{Post-test score (\%)}-\text{Pre-test score (\%)} \right]/\text{Pre-test score (\%)}$, which indicated knowledge acquired through the course.

UofT participants had higher pre-test scores than USP participants for both modules 1 and 2. While UofT participants showed significant knowledge enhancement for module 2, no substantial differences between pre- and post-test scores were noted for module 1. This may reflect the Canadian educational system's stronger emphasis on research, where trainees receive formal research training during undergraduate and medical education. However, gaps in statistical knowledge remain in the Canadian system, similar to deficiencies in research methodology and statistics in the Brazilian system. In this study, Canadian residents performed better in pre-tests than fellows/junior staff, likely due to prior exposure to research training during Canadian or US undergraduate education. For instance, UofT medical students undertake mandatory research courses during pre-clerkship years to develop skills in study design, research ethics, and statistical analysis. The Comprehensive Research Experience for Medical Students (CREMS) Program further provides mentorship opportunities for students considering careers as clinician-scientists [13]. Conversely, many Canadian fellows, the dominant category of fellows/junior staff participating in the course, typically receive education outside Canada, where formal research curricula may be absent, as in Brazil. This could explain why Brazilian residents and fellows showed no differences in pre-test scores for modules 1 and 2, despite fellows being senior trainees.

USP participants achieved average interval score changes exceeding 20% for both modules 1 and 2, in contrast to UofT participants. These encouraging results for Brazilian participants, combined with their positive feedback on the course's value, highlight the potential of such programs in developing countries to enhance the scientific profile of future radiologists. Additionally, a higher percentage of USP participants expressed interest in pursuing clinician-scientist careers compared to UofT participants. While Canadian participants' interval test score improvements were below expectations, Brazilian participants' improvements met expectations. This underscores the importance of research-oriented training for radiology trainees in emerging countries. Similar programs have demonstrated benefits in radiology research productivity, particularly in developing countries. For example, a study in a teaching hospital in Pakistan reported a significant increase in internationally published papers after implementing basic research skills training (p<0.001), from 77 in one year to 92 the next [14]. Studies in developed countries have also shown similar results [15], such as Diamond et al., where orthopedic surgery residents' test scores improved by a median of 8%, with significant differences between pre- and post-test scores (p<0.001) [16].

No MSc or PhD graduates participated in the present study. Prior research has shown that trainees with formal research training, such as MSc or PhD programs, publish more frequently during training and post-residency than those focused solely on clinical work [15]. Thus, providing incentives for further research training alongside residency could enhance the scientific expertise of future radiology leaders.

Several factors may explain the higher publication rates among Canadian trainees compared to Brazilian peers. Formal research training and institutional support provided to Canadian participants likely played a role, rather than the intervention alone, in influencing long-term academic productivity. Additionally, differing training expectations between the two countries' educational systems may have contributed. At UofT, radiology residents are required to submit at least one manuscript for publication consideration before graduating, while fellows must do so annually. In contrast, such requirements are not mandatory at USP. UofT's support system includes (1) one-on-one project supervision with weekly trainee-supervisor meetings, (2) early methodological review by committees, (3) periodic follow-up meetings, and (4) encouragement to attend internal and external research courses (e.g., Radiological Society of North America (RSNA) Writing a Competitive Grant Proposal [17], RSNA Clinical Trials Methodology Workshop [18], and Canadian Institutes of Health Research Young Investigators Forum [19]). Trainees are also required to present research findings locally and are encouraged to submit abstracts to external assemblies, with funding provided for accepted presentations. Conversely, USP at the time of the conduct of the course assessed in this study lacked a formal research curriculum, though travel grants and scholarships were available for RSNA attendees at the time the course was conducted in Brazil.

The higher IFs of journals where Canadian participants published may stem from differences in institutional expectations for supervisors. Canadian supervisors may receive stronger encouragement to publish in high-impact journals [20]. However, Brazilians' increase in journal IFs from timepoint 1 to timepoint 2 suggests that implementing a formal research curriculum could improve the quality of research output. Language barriers may also limit Brazilians' ability to publish in high-impact journals, which are predominantly in English.

Authorship order reflects cultural and institutional expectations [21]. In North America, first authorship typically indicates a trainee's leadership in all project phases, while supervisors serve as senior authors. Other systems may differ in assigning authorship. Despite these variations, similar first-author productivity was observed in this study, aligning with findings from a systematic review on gender inequality in pediatric radiology articles during COVID-19 [22]. In that study, whereas a tendency to a lower proportion of female first authors was noted pre-COVID-19 (p=0.075), there was no difference in male and female first authors of pediatric radiology publications in major journals post-COVID-19 (p=0.479).

No statistically significant gender differences were observed in publication numbers, IFs, or first-author publications in this study, suggesting equal research opportunities for the two cohorts analyzed. However, further studies with larger and more diverse populations are needed. Prior research in non-radiology fields conducted in developed countries has shown gender disparities in authorship [23-26], raising concerns about women's recognition in academia. These studies have shown that for papers in which three or more authors made equal contributions, more male authors than female authors had their names in the first position and more all-male than all-female author combinations were observed in several non-radiology fields. Potential contributing factors for the results of other studies towards the underrepresentation of women as first authors of publications, not confirmed in our study, include peer review, funding and protected time for research, collaborations with other investigators, and self-confidence among women researchers.

This study has limitations. Firstly, participant selection bias existed, as participation in the course was mandatory for Canadian trainees (who may not have been primarily research-oriented) and voluntary for Brazilian trainees (who may have had a stronger intrinsic interest in research), potentially influencing both engagement and outcomes. Notwithstanding, most participants of the two cohorts, regardless of having or not having aspirations towards a research-oriented career, rated the course's educational value and their increase in research knowledge highly (4 or 5). Secondly, the small sample size limited the generalizability of findings, and non-significant p-values should not be interpreted as evidence of no differences given the possibility of type 2 error. Thirdly, there is the possibility that some career aspirations reported by participants in their assessment may have been influenced by the course environment. Fourthly, the study did not adjust for potential confounding variables that may influence both short- and long-term outcomes, including baseline research experience, differences in institutional research infrastructure, availability of protected research time, and language barriers. These factors, particularly for participants from non-English-speaking environments, may have impacted both knowledge acquisition and publication productivity and should be considered when interpreting the results. Furthermore, the use of publication count and journal IF as surrogate markers of academic success has inherent limitations, as these metrics do not fully capture research quality, individual contribution, or broader academic impact. Finally, the study design does not allow for causal inference between the educational intervention and long-term publication outcomes, as multiple unmeasured structural and institutional factors may have influenced these results. Accordingly, causal inference between the educational intervention and long-term publication outcomes cannot be established based on the current study design. While some limitations were acknowledged by the authors in the paper, their impact on validity is underestimated. Nonetheless, although the results of this study are preliminary in nature, they point out a need for improvement of opportunities for research training in radiology in developing countries. Given that this study evaluated participants from only two countries, its generalizability may be limited. Future studies should expand this model to include multiple developing countries and diverse training environments to better assess the scalability and broader applicability of this educational intervention. Future research should evaluate online cross-cultural education, which expanded during the COVID-19 pandemic since the course was delivered in an in-person format.

Recommendations for implementing a radiology research program include early exposure to formal research training, beginning during undergraduate medical education, as emphasized by prior studies highlighting its importance for fostering future clinician-scientists [27]. Such training should be complemented by collaboration with experienced multidisciplinary research teams, which provides trainees with mentorship, methodological support, and exposure to diverse research perspectives. The development of "twin institution" programs can further enhance research capacity by enabling sustained remote educational collaboration between institutions in developed and developing countries, as proposed in this study [28]. Radiology trainees should be encouraged to attend workshops focused on research proposal development and grant writing to strengthen their ability to secure funding and lead independent projects. Creating structured opportunities to align research interests in developing countries with ongoing projects in developed nations may foster meaningful collaboration and accelerate the clinical translation of innovative imaging techniques. The findings of this study support the concept of scalable international training platforms that can be adapted and implemented across diverse healthcare systems, particularly in developing regions where formal research training remains limited. Future work should focus on expanding this model through multi-country implementation, incorporating virtual and hybrid delivery formats, and developing cross-cultural educational frameworks tailored to regional needs. Such approaches would enhance accessibility, promote global collaboration, and allow for broader validation of the effectiveness of structured research training programs.

## Conclusions

USP trainees demonstrated significant knowledge gains and a strong interest in research careers, underscoring the benefits of a radiology research program. While differences in long-term publication outcomes were observed between cohorts, these findings should be interpreted as associations rather than evidence of a direct causal effect of the intervention. These findings should therefore be considered hypothesis-generating and supportive of further investigation in larger, multi-institutional settings.

Leading institutions in developed and developing nations must enhance research education to fully utilize global resources. Future studies incorporating controlled designs and accounting for institutional and structural factors are needed to better understand the determinants of long-term academic productivity. Expanding these initiatives across multiple developing countries would strengthen their generalizability and contribute to reducing disparities in research capacity and academic productivity in radiology worldwide. This approach can elevate academic standards, create collaboration opportunities, and serve as a model for future initiatives in Latin America and other underserved regions. Sustained networking and knowledge dissemination should be key objectives for similar educational programs.

## Figures and Tables

**Figure 1:**
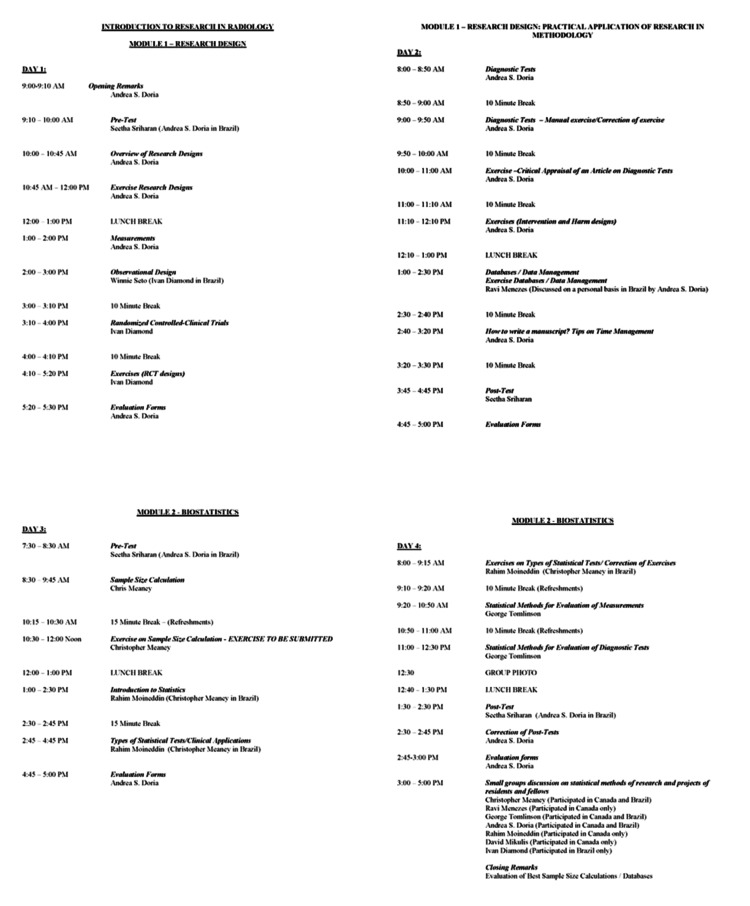
Course curriculum content

**Figure 2:**
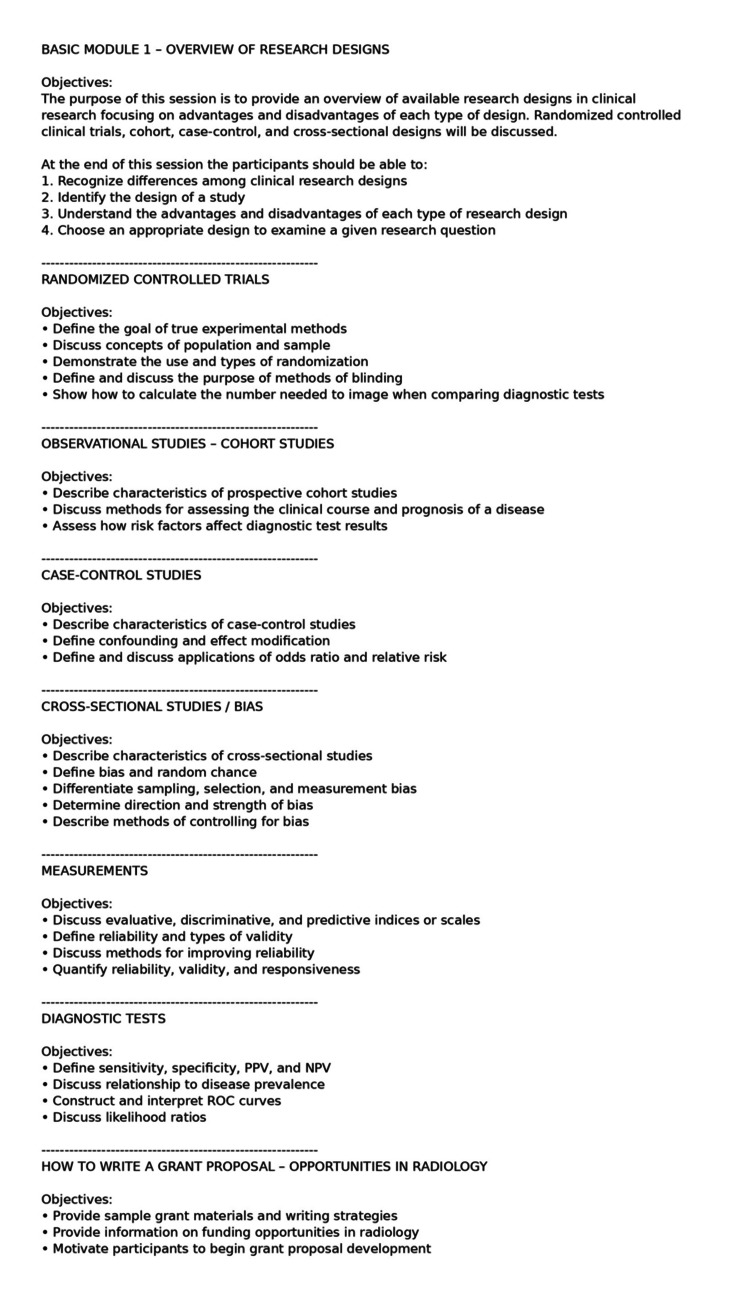
Objectives of individual educational sessions

**Figure 3:**
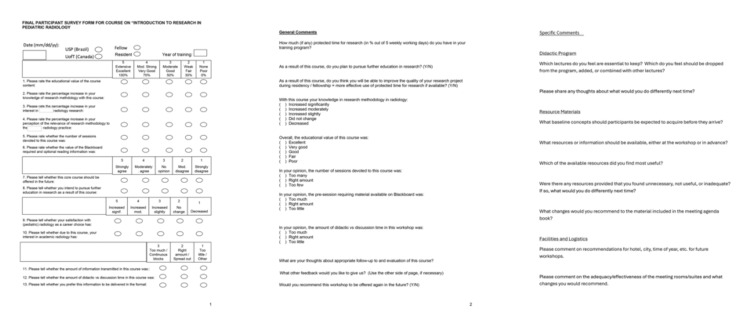
Final participant survey

## Appendices

Appendix A

Appendix B

Appendix C
